# *NCAPG* is differentially expressed during longissimus muscle development and is associated with growth traits in Chinese Qinchuan beef cattle

**DOI:** 10.1590/S1415-475738420140287

**Published:** 2015

**Authors:** Yu Liu, Xiaoyan Duan, Si Chen, Hua He, Xiaolin Liu

**Affiliations:** 1 College of Animal Science and Technology, Northwest A&F University, Yangling, Shaanxi, China; 2 HeBei North University, Zhang Jiakou, Hebei, China

**Keywords:** association analysis, growth traits, longissimus muscle, NCAPG expression, single nucleotide polymorphism

## Abstract

Based on RNA-seq analysis, we recently found that the bovine *NCAPG* (non-SMC condensin I complex, subunit G) gene is differentially expressed during development of the longissimus muscle. In the present study, we validated this result and, using quantitative real-time PCR analysis, identified two adjacent genes, LCORL and DCAF16, that are more abundant in fetal muscle tissue; further analysis of tissue-specific expression patterns indicated high abundance of *NCAPG* in muscle. Since no polymorphisms were detected in a previous study of Qinchuan cattle, we extended our investigation to examine the occurrence of single-nucleotide polymorphisms (SNPs) in the *NCAPG* gene. Three SNPs, *i.e.*, one located in the intron region (g47747: T > G), a synonymous mutation (g52535: A > G) and a missense mutation (g53208: T > G) that leads to a change in the amino acid of interest (pIle442Met), were detected in a population of Qinchuan beef cattle (n = 300). Association analysis showed that these SNPs were significantly associated with the growth traits of Qinchuan beef cattle. Our results indicate that the bovine *NCAPG* gene may be involved in the development of the longissimus muscle. These polymorphisms in the *NCAPG* gene may be useful for marker-assisted selection of optimal body size in Qinchuan beef cattle.

## Introduction

Optimal body size has been intensively investigated in beef cattle breeding and is considered to be a trait of high economic importance (Littlejohn *et al.*, 2011). Numerous genetic studies have sought to identify quantitative trait locus (QTL) or major genes associated with body size-related characteristics, such as growth and carcass traits.

In a genome-wide association study, Snelling *et al.* (2010) found a highly significant association between a chromosomal haplotype comprising the *NCAPG* (non-SMC condensin I complex, subunit G) gene and the body weight of cattle over time. The importance of the bovine *NCAPG* gene had previously been suggested by Setoguchi *et al.* (2009), who located a QTL for body or carcass weight in cattle (known as CW-2) in a 591-kb interval on bovine chromosome 6 (BTA6); they also identified a candidate causal variant in the *NCAPG* gene, *NCAPG*: c.1326T > G, responsible for the amino acid change p. Ile442Met. Additional studies (Eberlein *et al.*, 2009; Weikard *et al.*, 2010) that investigated the association of the *NCAPG*: c.1326T > G mutation with birth weight and body weight confirmed the role of this gene locus as the CW-2 QTL. These findings support the possibility that *NCAPG* regulates muscle growth in cattle and thereby influences muscle performance.

Based on RNA-seq analysis, we recently found that the *NCAPG* gene and its neighboring gene, *LCORL*, are both differentially expressed in longissimus muscle of fetal and adult Chinese Qinchuan beef cattle (He and Liu, 2013). This raises the possibility that *NCAPG* regulates muscle growth and thus influences the performance of Qinchuan beef cattle, the best-known native cattle breed in China. In the present study, we sought to identify important single nucleotide polymorphisms (SNPs) of the *NCAPG* gene and use this information for haplotype construction and association analysis. This investigation may contribute to our understanding of the role that *NCAPG* plays in the variation of cattle growth traits. Such knowledge could be relevant to improving beef cattle breeding practices in China.

## Materials and Methods

### *NCAPG* expression patterns in cattle

Quantitative real-time polymerase chain reactions (qRT-PCR) were used to examine the expression levels of *NCAPG* in heart, liver, spleen, lung and kidney samples from three adult Chinese Qinchuan cattle, and in longissimus muscle samples from three embryos at day 135 post-fertilization and three 30-month-old female adult cattle; the expression levels of *LCORL* and *DCAF16* in adult muscle samples were also examined. Tissue samples were obtained immediately after slaughter and were stored in liquid nitrogen until used. Total RNA was extracted with Trizol reagent (Ambion, USA). The quality (intactness) of the RNA was confirmed using a 2100 Bioanalyzer (Agilent, USA) and only samples with an RNA integrity number > 7 were used in subsequent analyses. One microgram of RNA from each sample was reverse-transcribed to cDNA using a PrimeScript RT reagent kit with gDNA Eraser (Takara, Japan). qRT-PCR was done with a CFX96 Real Time detection system (Bio-Rad) in triplicate using 2 SYBR^®^ Premix ExTaqTM II (TaKaRa, Japan). The data derived from the real-time PCR analysis were transformed using the formula 2^-^ΔΔCt (Livak and Schmittgen, 2001). For normalization, the *GAPDH* gene was used as an endogenous control. The primers used for qRT-PCR analysis were designed using Primer 5 software (PREMIER Biosoft International) and are shown in Table S1.

### DNA samples and phenotypic data

A pure-bred population of 300 Qinchuan beef cattle (30 ± 2 months of age, bullocks) was used in this study to identify mutations in the bovine *NCAPG* gene. The cattle were from a single farm, reared under identical conditions and fed the same diet. The calves were weaned to six months of age on average and were then raised to slaughter on a diet of corn and corn silage.

Genomic DNA from the 300 Qinchuan cattle was isolated from 2% heparin-treated blood samples and stored at −80 °C as standard procedure (Sambrook and Russell, 2002). The DNA was diluted to 50 ng/μL in ddH_2_O and stored at −20 °C until further analysis.

The traits used to describe cattle body size were body height (BH, cm), body length (BL, cm), hip width (HW, cm), body weight (BW, kg) and carcass weight (CW, kg). Carcass weight was measured right after slaughter while the other parameters were measured right before slaughter. All of the traits were measured according to the GB/T17238-1998 criterion for the Cutting Standard of Fresh and Chilled Beef in China (China Standard Publishing House). All of the experimental procedures were performed according to the terms of the authorization granted by the Chinese Ministry of Agriculture.

### SNP detection and genotyping

DNA sequencing was used to identify sequence variations in the *NCAPG* gene. Triplicate samples of DNA from each animal were used as the template to amplify the different regions of *NCAPG*. The primers used for amplification of the *NCAPG* gene were designed from a published gene sequence (GenBank accession number: AC_000163.1) using Primer 5 software and are shown in Table S2. The PCR amplifications were done in a final volume of 15 μL containing 50 ng of genomic DNA as the template and 10 mM Tris-HCl buffer (pH 8.8) with 50 mM KCl, 0.2 μM of each primer, 200 μM dNTP, and 0.5 U of *Taq* DNA polymerase (MBI Fermentas, USA). The PCR conditions were as follows: after an initial denaturation for 5 min at 95 °C, the amplicons were generated using 35 cycles of 30 s at 94 °C, 30 s at an optimized annealing temperature (Table S2) and 45 s at 72 °C, and a 10 min final extension step at 72 °C. The products were sequenced in both directions using an ABI PRISM 3730 DNA analyzer (Sango, Shanghai, China). The sequences were analyzed using DNASTAR software (version 7.0).

PCR-RFLP and forced PCR-RFLP were used to genotype the cattle. The primer information and restriction enzymes (REs) are shown in Table S2. The PCR products were digested with their respective REs at distinct temperatures and the digested products were then separated by electrophoresis on 3% agarose gels.

### Statistical analysis

The population genetic diversity parameters, including allele and genotype frequencies, effective number of alleles (Ne), heterozygosity (He), homozygosity (Ho), Hardy-Weinberg equilibrium (HWE) and polymorphism information content (PIC) were estimated using Popgen32 software. The linkage disequilibrium (LD) structure of three loci, as measured by D) and r2, was determined using HAPLOVIEW (Barrett *et al.*, 2005; Huang *et al.*, 2013). The PHASE program (Stephens *et al.*, 2001) was used to calculate the individual haplotypes. General linear models (GLMs) were generated with SPSS software (ver. 16.0) to investigate the association of *NCAPG* mutations with growth and carcass traits (Holzer and Precht, 1992; Huang *et al.*, 2011) using the model: Yij = μ + ai + eij, where Yij is the trait value observed for animal j and genotype i, μ is the overall population mean, ai corresponds to the fixed effect of genotype i, and eij is the residual error. A p value < 0.05 was considered to be significant. For a more detailed analysis of the results, we corrected the p values using the Bonferroni correction (a = 0.05/3) to account for multiple tests and obtain more robust comparisons.

## Results

### *NCAPG* expression levels

**Figure 1:**
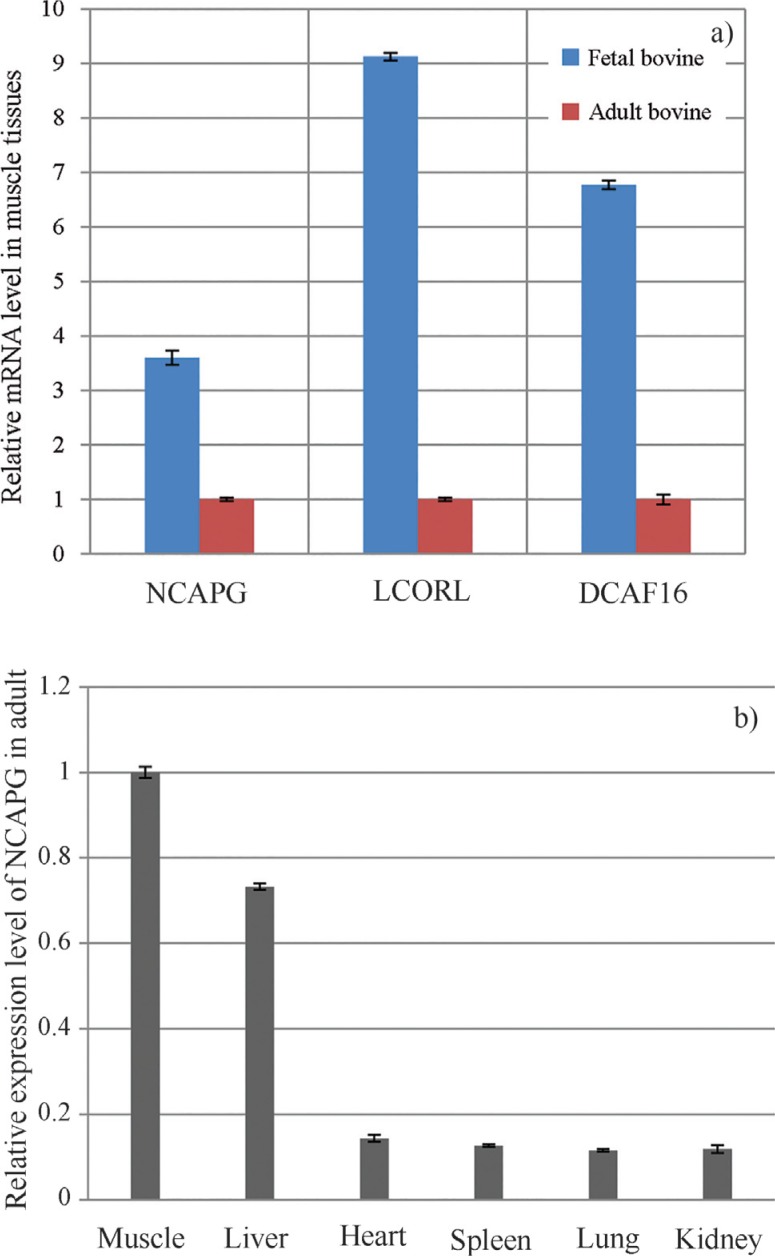
A. Expression levels of the *NCAPG*, *LCORL* and *DCAF16* genes in fetal and adult muscle tissue, B. Tissue-specific expression patterns of the *NCAPG* gene in adult cattle. The columns represent the means ± SEM (n = 3).

Figure 1A shows the levels of *NCAPG* gene expression during development of the longissimus muscle as assessed using qRT-PCR. *NCAPG* was up-regulated in fetal muscle compared with adult muscle. Among the various tissues screened, *NCAPG* expression was greatest in muscle followed by liver; low expression was seen in other organs (heart, kidney, lung and spleen) (Figure 1B). As with *NCAPG*, the expression of *LCORL* and *DCAF16* (two neighboring genes) was also much greater in fetal muscle compared to adult muscle.

### SNP detection and diversity analyses

**Figure 2:**
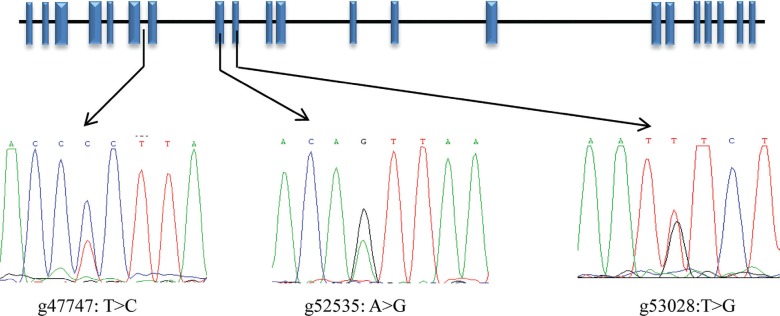
Schematic representation of the *NCAPG* gene showing the location of the three SNPs. Blue blocks are gene exons.

Three SNPs (g47747: T > C, g52535: A > G and g53208: T > G) were detected and are shown in Figure 2. The SNP g47747: T > C was located in intron six, g52535: A > G was a synonymous mutation located in exon eight, and g53208: T > G was a missense mutation leading to the amino acid change p. Ile442Met in the NCAPG protein.

The SNPs were successfully genotyped using PCR-RFLP and force PCR-RFLP, as shown in Figure S1. Distinct genotypes were defined by distinct banding patterns. The individuals with g47747: T > C were genotyped using PCR-RFLP. Digestion of the resulting 495-bp PCR fragment of *NCAPG* with *Eco81*I resulted in fragments with band lengths of 302 and 193 bp for individuals with the CC homozygous genotype, 495, 302 and 193 bp for TC heterozygotes, and 495 bp for TT homozygotes. PCR-RFLP was also used to genotype the individuals with g52535: A > G. Digestion of the resulting 406-bp fragment with *VspI* resulted in fragment lengths of 296 and 110 bp for AA homozygotes, 406, 296 and 110 bp for TC heterozygotes, and 406 bp for TT homozygotes. Forced PCR-RFLP was used to genotype the individuals with g53208: T > G. Digestion of the resulting 141-bp fragment with *XapI* resulted in fragment lengths of 122 and 19 bp for TT homozygotes, 141, 122 and 19 bp for TG heterozygotes, and 141 bp for GG homozygotes.

The allele and genotype frequencies and the genetic diversity parameters (Ho, He, Ne and PIC) of the three SNPs are shown in Table 1. The three loci identified in Qinchuan cattle were in Hardy-Weinberg equilibrium (p > 0.05). Our results suggest that Qinchuan cattle are in equilibrium with regard to artificial selection, migration and genetic drift. The PIC values ranged from 0.3541 to 0.3688, indicating an intermediate genetic diversity for the *NCAPG* gene in the population analyzed.

**Table 1:** Genetic diversity parameters for the SNPs detected in this study.

Mutations	Genotype	Frequencies	Allelic frequencies	χ^2^ test	Ho	He	Ne	PIC
g47747: T > C	TT (n = 97)	0.3233	T(0.5783)	p > 0.05	0.5123	0.4877	1.95	0.3688
	TC (n = 153)	0.51	C(0.4217)					
	CC (n = 50)	0.1667						
g52535: A > G	AA (n = 37)	0.1233	A(0.3583)	p > 0.05	0.5471	0.4529	1.83	0.3541
	AG (n = 141)	0.47	G(0.6417)					
	GG (n = 122)	0.4047						
g53208: T > G	TT (n = 117)	0.39	A(0.6167)	p > 0.05	0.5272	0.4728	1.897	0.3610
	TG (n = 136)	0.4533	G(0.3833)					
	GG (n = 47)	0.1567						

He - gene heterozygosity, Ho - gene homozygosity, Ne - effective number of alleles, PIC - polymorphism information content. χ^2^ test: Hardy-Weinberg equilibrium (HWE) χ^2^ value (p > 0.05 indicates equilibrium and p < 0.05 indicates disequilibrium).

### Linkage disequilibrium and haplotype analysis

The linkage disequilibrium between the polymorphism pairs and the haplotype structure of the *NCAPG* gene are summarized in Tables 2 and 3, respectively. The linkage disequilibrium between the three SNPs was expressed as D) and r^2^ using HAPLOVIEW. The values of D) ranged from 0.184 to 0.323, and the r^2^ values ranged from 0.017 to 0.05. These results indicated that the three SNPs were in low linkage disequilibrium. The haplotype structure analysis was done using PHASE. Six haplotypes were identified in the population. Hap11 (-TGT-) had the highest haplotype frequency (29%) and Hap12 (-CAG-) had the lowest haplotype frequency (5%).

**Table 2:** Linkage equilibrium parameters estimated for the three *NCAPG* gene SNPs detected in this study.

SNP	g47747:T > C	g52535: A > G	g53208: T > G
g47747:T > C		D’ = 0.184	D’ = 0.217
g52535: A > G	r^2^ = 0.017		D’ = 0.323
g53208: T > G	r^2^ = 0.023	r^2^ = 0.05	

**Table 3:** Haplotype frequencies for the three *NCAPG* gene SNPs detected in Qinchuan beef cattle.

Haplotype	Position of sequence variants			Frequency in population
	g47747: T > C	g52535: A > G	g53208: T > G	
Hap1	T	A	T	0.1667
Hap2	T	G	T	0.29
Hap 3	T	G	G	0.2067
Hap 4	C	G	T	0.1266
Hap 5	C	G	G	0.16
Hap 6	C	A	G	0.05

### Association study

The association analysis focused mainly on the statistical correlation between genetic markers (SNPs) and traits (Botstein and Risch, 2003). In particular, we analyzed the associations of the three SNPs with growth traits in Qinchuan cattle. Table 4 summarizes the results of the association analyses between individual markers and growth traits.

**Table 4:** Effects of *NCAPG* genotypes on growth and carcass traits in Qinchuan beef cattle.

SNP	Genotype	N	BH (cm)	BL (cm)	HW (cm)	BW (kg)	CW (kg)
g47747: T > C	TT	97	141.26 ± 3.575	154.143 ± 3.913^a^	45.958±0.803	492.191 ± 18.372	270.176 ± 5.237
	TC	153	138.74 ± 2.534	148.217 ± 2.927^ab^	47.344 ± 0.568	493.761 ± 16.282	265.161 ± 6.957
	CC	50	134.36 ± 2.782	142.556 ± 4.237^b^	46.571 ± 1.051	496.221 ± 15.237	263.652 ± 6.715
	*P* 1		0.287	0.039	0.278	0.397	0.161
g52535: A > G	AA	37	141.528 ± 3.846	151.417 ± 4.231^a^	46.586 ± 1.425	495.311 ± 18.281	274.817 ± 10.275^a^
	AG	141	136.832 ± 2.455	146.694 ± 3.238^ab^	47.254 ± 0.578	494.798 ± 20.281	262.241 ± 9.275^b^
	GG	122	138.256 ± 4.014	139.222±6.252^b^	46.856 ± 0.693	498.061 ± 15.291	266.245 ± 5.275^ab^
	*P* 1		0.428	0.041	0.716	0.531	0.041
g53208: T > G	TT	117	137.075 ± 2.626	136.728 ± 4.693^b^	44.676 ± 1.412^b^	493.761 ± 19.281	265.821 ± 4.281^b^
	TG	136	139.9814 ± 3.128	150.871 ± 6.236^ab^	46.187 ± 0.842^ab^	505.761 ± 15.169	269.981 ± 5.298^ab^
	GG	47	141.265 ± 4.134	156.297 ± 3.551^a^	47.500 ± 0.772^a^	497.216 ± 20.285	278.138 ± 8.151^a^
	*P* 1		0.875	0.017	0.036	0.415	0.031

BH - body height, BL - body length, BW - body weight, CW - carcass weight and HW - hip width. Data expressed as the mean ± SEM. Values with different superscripts (a, ab and b) within the same column differ significantly (p < 0.05).

1 Probability of the F-test for genotype effect.

In agreement with our previous results for g47747: T > C, the animals with the TT genotype had longer bodies than those with the CC genotype (p < 0.05). In contrast, the analysis of g52535: A > G showed that individuals with the AA genotype tended to have longer bodies and heavier carcasses than those with the GG genotype (p < 0.05). The analysis of g53208: T > G revealed that individuals with genotype GG had significantly greater body length, hip width and carcass weight compared with AA homozygote (p < 0.05); the association between g53208: T > G and body length remained significant after the Bonferroni correction, which suggested that this was the most important association detected in our analysis.

## Discussion

The bovine *NCAPG* gene is located on chromosome BTA6 and has attracted much attention because of its effect on cattle growth traits. RNA-seq and qRT-PCR analyses have shown a high abundance of *NCAPG* transcripts in muscle compared to other tissues, with greater abundance in fetal compared to adult muscle. The greater expression of *NCAPG* in fetal muscle suggests that this gene may play an important role in early muscle development. Metzger *et al.* (2013) investigated the relative expression levels of *LCORL* and its two neighboring genes, *NCAPG* and *DCAF16*, and demonstrated a significant association of the relative *LCORL* expression levels with horse size. Lindholm-Perry et al. (2014) also identified a relationship between *NCAPG* expression in LD (linkage disequilibrium) muscle and average daily gain for cows. As shown here, *NCAPG*, *LCORL* and *DCAF16* were all expressed at low levels in adult muscle tissue. This finding suggests that *NCAPG* expression may be associated with the development of bovine muscle, although further research is required to elucidate the causal mechanism.

Since we had previously identified no SNPs in the *LCORL* gene of Qinchuan beef cattle, in the present study we focused on the *NCAPG* gene. Three SNPs were detected by sequencing: an intron mutation (g47747: T > C), a synonymous mutation (g52535: A > G) and a missense mutation (g53208: T > G) that leads to the amino acid change p. Ile442Met in the *NCAP*G protein. The ancestral population structure, which is reflected in the distribution of haplotypes, can occasionally provide greater power than single-marker analysis for studying genetic diseases and trait associations (Akey *et al.*, 2001). As shown here, six haplotypes were present at varying frequencies. One explanation for this variation in haplotype frequency is that new mutants are derived from several common haplotypes and common high-frequency haplotypes have persisted in the population for a long time (Posada and Crandall, 2001).

In this study, meaningful associations were found between SNPs and growth traits. Based on our statistical analysis, individuals with the TT genotype at locus g47747: T > C, the AA genotype at locus g52535: A > G and the GG genotype at locus g53208: T > G could be selected to obtain the optimal body size. The SNPs g47747: T > C and g52535: A > G are silent mutations that do not change the amino acid composition of the expressed protein but are nonetheless associated with the growth traits of Qinchuan cattle. In agree with this, there have been several reports on the effects of silent mutations on cattle development. Three silent mutations of the bovine *GL*13 gene are associated with body weight at birth and at six months of age in Nanyang cattle (Huang *et al.*, 2013). Silent mutations in the bovine *INSIG*1 gene have also been associated with growth traits in Qinchuan beef cattle (Liu *et al.*, 2012). The mechanism underlying the association between silent mutations and growth traits in beef cattle has yet to be determined.

The g53208: T > G SNP is a missense mutation that encodes a change from Ile to Met (p. Ile442Met). In a previous study, Dej *et al.* (2004) found that the *NCAPG* gene encodes a protein of the condensin I complex that has an important function in regulating mitotic cell division. Additionally, Seipold *et al.* (2009) previously reported that an *NCAPG* mutation predominantly affects the highly proliferative progenitor cells of the zebrafish neural retina. This mutation in cattle may also participate in this biological process but its mechanism needs further research. Previous investigations have focused on the association of this missense mutation with the phenotypic traits of cattle. Weikard *et al.* (2010) found that this SNP in *NCAPG* was associated with the time course of average daily gain in Japanese Black and Charolais German Holstein populations. Eberlein *et al.* (2009) found that this SNP was associated with birth weight in a Charolais German Holstein cross population. In our study, this missense mutation was significantly associated with body length, hip width and carcass weight, a finding in general agreement with previous reports. These results suggest that the g53208: T > G SNP is significantly associated with growth traits of numerous cattle breeds, although the mechanism underlying this relationship requires further research. Interestingly, this SNP is significantly associated with body length, hip width and carcass weight, but not body weight. Nevertheless, the GG genotype tended to have a greater body weight than the TT genotype, although this difference may reflect the small sample size in our research. An association analysis with a larger sample group should yield more robust results.

In conclusion, the results of this study indicate a significant difference in the expression of the *NCAPG*, *LCORL* and *DCAF16* genes in fetal and adult bovine longissimus muscle, which suggests that they may be involved in muscle development. Three SNPs in *NCAPG* were associated with bovine growth traits. Together, these findings suggest that *NCAPG* gene polymorphisms could be potentially useful genetic markers for breeding programs aimed at improving Qinchuan beef cattle. However, further studies are needed to establish the functional effects of the various alleles and the mechanisms involved. Such information will improve our understanding of the role of *NCAPG* in the genetic regulation of cattle growth.

## Supplementary Material

The following online material is available for this article:

Figure S1 PCR-RFLP and force PCR-RFLP amplification products for the *NCAPG* gene.

Table S1 Primers used for qRT-PCR.

Table S2 Primer sequences and PCR conditions used for amplifications and RFLP analysis.

This material is available as part of the online version of this article from http://scielo.br/gmb.
