# Supplementary material for: NCAPG is differentially expressed during longissimus muscle development and is associated with growth traits in Chinese Qinchuan beef cattle
Source: Genet Mol Biol. 2015 Oct-Dec;38(4):450–6. doi: 10.1590/S1415-475738420140287 (PMC4763315; doi:10.1590/S1415-475738420140287)
Supplement: Figure S1 [file 1415-4757-gmb-38-04-450-s001.pdf]

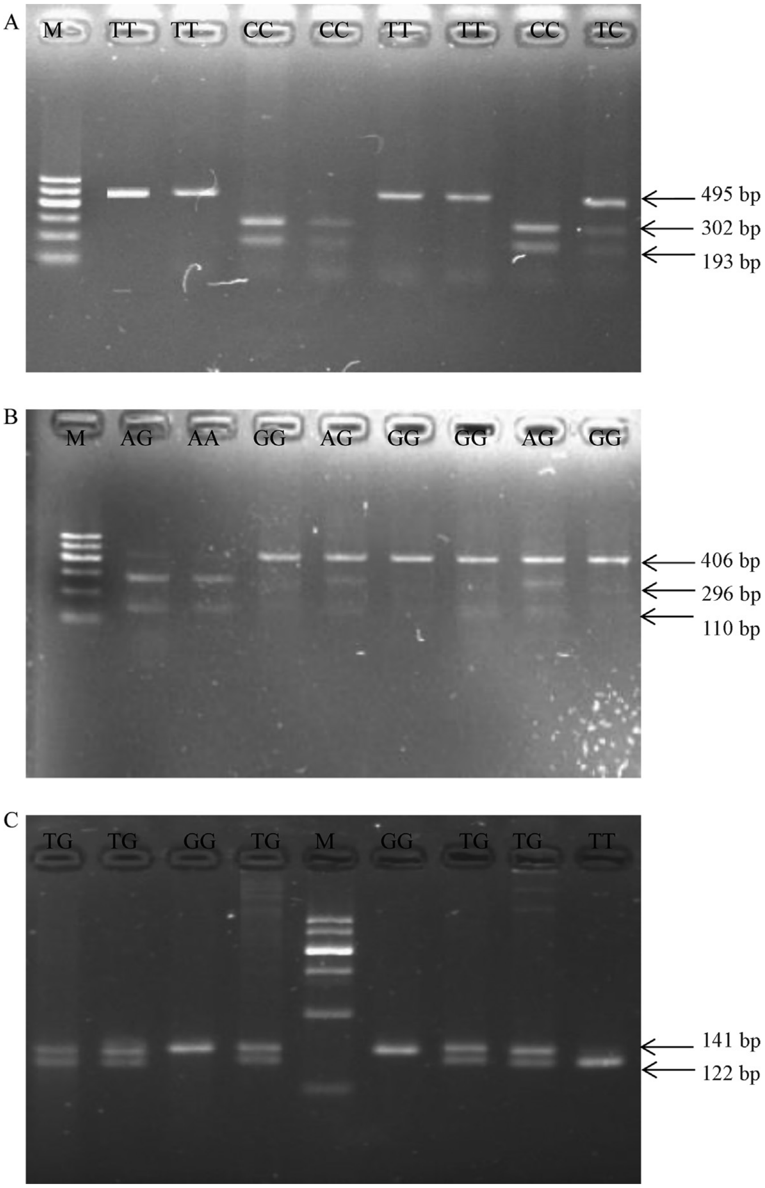

Figure S1 - PCR-RFLP and force PCR-RFLP amplification products for the *NCAPG* gene. (A) g47767: T > G genotype: TT = 495 bp, TC = 495 bp + 302 bp + 193 bp, CC = 302 bp + 193 bp, (B) g52535: A > G genotype: AA = 296 bp + 110 bp, AG = 406 bp + 296 bp + 110 bp, GG = 406 bp, and (C) g53208: T > G genotype: TT = 122 bp + 19 bp, TG = 141 bp + 122 bp + 19 bp, GG = 141 bp. All amplification products were run on 3% agarose gels. However, the 40 bp and 19 bp DNA fragments are difficult to seen on these gels. M - DNA molecular size markers (600, 500, 400, 300, 200, 100 bp).
