# Supplementary material for: NCAPG is differentially expressed during longissimus muscle development and is associated with growth traits in Chinese Qinchuan beef cattle
Source: Genet Mol Biol. 2015 Oct-Dec;38(4):450–6. doi: 10.1590/S1415-475738420140287 (PMC4763315; doi:10.1590/S1415-475738420140287)
Supplement: Table S1 [file 1415-4757-gmb-38-04-450-s002.pdf]

Table S1 - Primers used for qRT-PCR.

Primer	Primer sequence (5'-3')	Annealing temperature (°C)	Fragment size (bp)	Reference seq
NC-qRT	F: GCGGGCAGTGTTATCGTG<br>R: AGCCAGCTTTCTGACAGTTTCT	60	97	NM_001102376
LC-qRT	F: GGGACCTCTCTCCTTTGTGT<br>R: CCTGGGCTGCTTATTATCTT	60	104	NM_001192357.1
DC-qRT	F: GCCTCCTCTGGCTTCTTG<br>R: GTGGGGGGTGGCTCTACT	60	148	NM_001078147.1
GA-qRT	F: ATCATCTCTGCACCTTCTGCCGAT<br>R:TAAGTCCCTCCACGATGCCAAAGT	60	166	
