# Supplementary material for: NCAPG is differentially expressed during longissimus muscle development and is associated with growth traits in Chinese Qinchuan beef cattle
Source: Genet Mol Biol. 2015 Oct-Dec;38(4):450–6. doi: 10.1590/S1415-475738420140287 (PMC4763315; doi:10.1590/S1415-475738420140287)
Supplement: Table S2 [file 1415-4757-gmb-38-04-450-s003.pdf]

Table S2 - Primer sequences and PCR conditions used for amplifications and RFLP analysis.

Primer	Primer sequences (5’-3’)	SNP	Anealing temperature (°C)	Mg^{2+} concentration (mM)	Fragment size (bp)	Restriction enzyme
P1	F: AGGGGAACTTGGTTCACCTA<br>R: ATGCATGCTGTTGGACTTTT	g47747:T>C	56.4	2.0	495	*Eco81I*
P2	F: TTGAATCAAGCCAGTAAGTT<br>R:GACTGTATTTTAACAAAGACTATT	g52535: A>G	54.2	1.5	406	*Vsp I*
P3	F: CTACCACACCAATATCCC**A**AAT<br>R: ACTCAATGTTATTGACCAAAAT	g53208: T>G	58.9	2.0	141	*Xap I*

The nucleotide in bold (primer P3) mismatches, allowing the use of selected restriction enzymes to discriminate sequence variations.

SNP – single nucleotide polymorphism.
